# Editorial: Functional and Structural Connectomics of Mood Disorders

**DOI:** 10.3389/fpsyt.2019.00202

**Published:** 2019-04-16

**Authors:** Amit Anand

**Affiliations:** Center for Behavioral Health, Cleveland Clinic Lerner School of Medicine, Cleveland, OH, United States

**Keywords:** depression, MDD, fMRI, DTI, resting state connectivity, functional connectivity, structural connectivity, connectome

Despite decades of research, reliable biomarkers for diagnosis and treatment of mood disorders are yet to be found. Brain imaging studies of mood disorders have evolved from studying regional brain abnormalities to investigating the whole brain connectome. However, clinical application of reported connectome metrics remains elusive. Several reasons underlie the lack of progress in this area. These include the reliance of connectome studies on investigating simple pair-wise connections between brain regions rather than on studying network properties and patterns; investigation of a limited number of fronto-temporal brain regions such as the anterior cingulate cortex and the amygdala implicated in early studies of mood disorders; and lack of analytic tools to develop accurate classifiers using large scale complex data. Furthermore, most studies do not account for confounding factors such as age, gender, and comorbid conditions. In this special topic collection of articles, investigators report results from a variety of studies of the brain connectome in major depressive disorder (MDD) that address some of the limitations of studies done so far.

Lu et al. report results of a diffusion tensor MRI study in early-stage MDD. They investigated structural connectome graph theory (GT) metrics abnormalities and found that MDD group had a decrease in the small-worldness property within the global brain network. Furthermore, using network-based statistics (NBS), they report weakness in the cortical (orbitofrontal cortex)-subcortical (thalamus/hippocampus) network in MDD. The finding that these structural connectome abnormalities are present even in early stage first-episode drug-naïve subjects suggests that they may be trait related biomarkers of vulnerability for development of MDD.

Anand et al. report findings from a 3-T fMRI study to investigate resting state functional connectivity of the dorsal raphe nucleus (DRN)(serotonin neurons) and the ventral tegmental nucleus (VTA)(dopamine neurons), with the cortical and subcortical regions. Unlike cortical and subcortical areas, ROIs for brainstem nuclei for the human brain are not available in most imaging analysis software. Using a careful anatomical approach they constructed regions of interest (ROI) for DRN and VTA. The results of the study show a significant decrease in DRN-cingulate cortex connectivity in medication-free young MDD subjects compared to healthy controls and a correlation of DRN amygdala/hippocampus complex connectivity with the severity of depressive symptoms. VTA connectivity with the parietal cortex was related to a reduction in depression severity after 8 weeks of treatment with an antidepressant. Further work is needed to study brainstem nuclei connectivity due to their central role in monoaminergic and cholinergic neurotransmission as well as in neuronal systems involved in arousal.

Using a machine-learning approach to predict antidepressant treatment response, Leaver et al. report that blood flow measured with arterial spin labeling (ASL) and GT properties of low-frequency BOLD fluctuations can predict antidepressant response to ECT at baseline. They report that imaging features selected by a support-vector machine model, to distinguish between Responders vs. Non-responders, consistently involved a left frontoparietal network as well as temporal-occipital and temporal-subgenual cingulate networks. Classification accuracy was not high [sensitivity (54–64%) or specificity (55–75%)], but connectivities within these networks correlated with change in depression scores after ECT treatment. The results of the study underscore the promise of machine-learning tools for diagnostic classification and treatment prediction.

It has been increasingly recognized that the brain functional connectome is not static and is continually changing. In a sophisticated analysis of dynamic functional connectivity in a large sample of Chinese Han subjects, Zhi et al. report Independent Component Analysis (ICA) and GT metric differences between MDD subjects and healthy controls (HC). Besides finding differences in connectivity between MDD and healthy HC subjects, they report that MDD subjects spend more time in states with weak global connectivity compared to HCs. Functional connectivity strengths between ICA components within each state correlated with changes in depression scores. This report calls to attention the importance of keeping the dynamic nature of the functional connectome in mind while exploring biomarkers of illness severity and treatment response in mood disorders.

Finally, confounding effects of comorbid conditions such as sleep disturbance frequently seen in mood disorders need to be addressed while studying the connectome. Klumpp et al. report that regardless of diagnosis of depression or anxiety disorder, poor self-reported sleep quality correlates with abnormal amygdala connectivity. Poor reported sleep correlated negatively with left amygdala-subgenual connectivity and positively with cerebellar-temporal gyrus resting state functional connectivity. The findings of this study suggest that sleep-related abnormalities seen in psychiatric disorders may be transdiagnostic and it is important to control for them in data analysis.

The importance of age and gender effects on connectome metrics is increasingly recognized. Using a novel approach, Conrin et al. report that gender differences in the hierarchical modularity of the functional connectome changes with age. Differences between males and females start appearing in the 26–30 years age group and become more significant by 31–35 year age. This change in difference over time was primarily derived from changing hierarchical modular structure in males while in females the modular structure remained more stable. Anxiety and inattention symptomatology correlated with a Task Positive Network (TPN) in males. The findings of this study suggest that the resting or basal configuration of the functional connectome may need to be re-conceptualized with more granularity and that gender differences need to be studied as a function of age.

The reports contributing to the special topic of the connectome in MDD exemplify the challenges involved in the study of the connectome in mood disorders and other neuropsychiatric illnesses. On the one hand, simple models of the connectome such as pair-wise regional connectivity are not able to provide important information regarding network-level patterns and properties. On the other hand, studying network properties which are dynamic and which can be affected by sleep, age and gender requires highly complex analysis. Data reduction methods such as ICA ad GT can provide tools to conduct such analysis, but the consistency of results from these advanced methods needs to be validated and replicated. Finally, machine-learning and deep learning tools need to be developed to the point that they can provide high levels of classification accuracy so that connectomic metrics can be used in clinical practice.

## Author Contributions

The author confirms being the sole contributor of this work and has approved it for publication.

### Conflict of Interest Statement

The author declares that the research was conducted in the absence of any commercial or financial relationships that could be construed as a potential conflict of interest.

